# Antibacterial and Immunoregulatory Effects of Metformin against *Helicobacter pylori* Infection in Rat Model

**DOI:** 10.1155/2023/5583286

**Published:** 2023-11-21

**Authors:** Hassan Valadbeigi, Saeed Khoshnood, Babak Negahdari, Mohd Azmuddin Abdullah, Mohammad Hossein Haddadi

**Affiliations:** ^1^Clinical Microbiology Research Center, Ilam University of Medical Sciences, Ilam, Iran; ^2^Department of Medical Biotechnology, School of Advanced Technologies in Medicine, Tehran University of Medical Sciences, Tehran, Iran; ^3^SciCo Science and Technology Center, Mody Rd 62. Yau Tsim Mong District, Kowloon, Hong Kong SAR, China

## Abstract

**Introduction:**

*Helicobacter pylori* (*H. pylori*) induces gastritis by stimulating Th17 cells and related cytokines. The aim of our study was to investigate the synergistic effect of metformin with amoxicillin as an antibiotic in inhibiting *H. pylori* and modulating the immune response in a rat model.

**Methods:**

Forty-five male Sprague-Dawley rats were divided into seven groups and infected with *H. pylori*. Over the course of 14 days, all animals were treated with metformin and amoxicillin alone and in combination. The antibacterial activity of metformin was evaluated by growth curves and colony counts. The immunoregulatory effect on Treg/Th17 balance was assessed by flow cytometry, and the cytokine profile of IL-17A, IL-1*β*, IL-6, IL-8, TGF-*β*, and IL-10 was determined by ELISA. The effect of metformin on gene expression of *cagA* and IL-8 was investigated by RT-PCR. Pathological changes were assessed by hematoxylin and eosin (H&E) staining and immunohistochemical (IHC) staining.

**Results:**

Metformin showed weak antibacterial activity against clinically isolated *H. pylori*. However, the combination of metformin and amoxicillin (AMX) showed strong synergistic antibacterial activity (*Σ*FIC = 0.24). Compared with AMX, metformin reduced inflammation and tissue damage but resulted in increased bacterial growth. During metformin administration, both TGF-*β* levels and Treg cells increased dramatically (*P* = 0.002). In synergy with AMX, metformin decreased the effective dose of antibiotic to eradicate *H. pylori*.

**Conclusions:**

The combination of metformin with potential antibiotics such as AMX had a positive effect on the relief of *H. pylori*-related inflammation by inducing Treg cells while successfully eliminating *H. pylori*.

## 1. Introduction

Metformin, a dimethylbiguanide, inhibits gluconeogenesis, improves glucose uptake into skeletal muscle, and reduces insulin resistance [1]. In mouse models of autoimmune disease, metformin inhibits the growth of T helper 17 (Th17) cells and the production of the corresponding cytokines, while increasing the expression of p-AMPK and forkhead box protein 3 (FOXP3) of Treg cells. Metformin reduces the expression of proinflammatory cytokines in treated HT-29 cells in a dose-dependent manner [2, 3].

Metformin's antibacterial activity is still unclear. Several studies have demonstrated the antibacterial effect of adjuvants against Gram-positive and Gram-negative bacteria. In addition, it is applied to various biological mechanisms, including alteration of the immune response to infection, potentiation of antibiotic activity, and the ability to act by destroying the outer membranes and efflux pumps of bacteria. For example, Novita et al. reported that metformin has the potential to increase the efficacy of anti-TB by enhancing autophagy [4]. In another study by Liu et al., metformin restores antibiotic susceptibility through intracellular accumulation of doxycycline in tetracycline-resistant *Escherichia coli* [5]. In addition, metformin was found to target the outer membrane of Gram-negative bacteria, thus inhibiting the growth of *Klebsiella pneumoniae* [6, 7].

*Helicobacter pylori* (*H. pylori*) is a microaerophilic, spiral-shaped bacterium that causes gastric cancer. As such, it is the only bacterium recognized by the World Health Organization as a class I carcinogen [8]. In preclinical studies, metformin has been shown to prevent the growth and colonization of *H. pylori* in mice [9]. There is evidence that metformin can temporarily halt the progression of gastric pathogenesis caused by *H. pylori* infection during two weeks of therapy [8].

In addition to the imbalance between Th1 and Th2 cells, regulatory T cells (Treg) and Th17 responses also play an important role in the pathogenesis of *H. pylori* [10, 11]. *H. pylori* triggers Treg-mediated immunologic responses and associated cytokines such as TGF-*β* and IL-10, with the goal of evading the immune system [12]. Inflammation and IFN-*γ* production are much higher in mice with *H. pylori* infection. Consequently, transplanted T cells after removal of Treg cells resulted in higher pathogenicity of bacteria than transferred T cells plus Treg cells [13]. As a consequence of *H. pylori* infection, different subsets of T cells become active. Proper activation of specific Th1 and Th17 cells is required for clearance of *H. pylori*. Gastric ulcers and gastritis may also occur as a result of uncontrolled activation of Th17 and/or Th1 cells [14, 15]. Alternatively, gastric malignancies associated with *H. pylori* may be triggered by abnormal Th2 and Treg cell responses [16].

The aim of this study is to gain a better understanding of the antibacterial and immunoregulatory effects of metformin on aspirin-induced gastric ulcers and *H. pylori* infections in rats. In this study, we show that metformin in combination with amoxicillin (AMX) has adjuvant antibacterial effects. When we examined Th17 and Treg cells, we found that metformin enhanced immunosuppression by inducing Treg cells and increasing their cytokine production, whereas it reduced Th17 cells and proinflammatory cytokines in the *H. pylori*-infected rat model. The results of our study also showed that metformin reduced the effective dose of amoxicillin required for *H. pylori* eradication and accelerated gastric ulcer healing. Our data suggest a significant effect of adjuvant antibacterial therapy on *H. pylori* eradication and reduction of associated complications such as gastric ulcers.

## 2. Materials and Methods

### 2.1. Reagents

Metformin (hydrochloride) and AMX were purchased from Sigma-Aldrich (Germany). The following monoclonal antibodies (mAbs) were used for flow cytometric analysis: anti-CD4 FITC, anti-Foxp3 PE, anti-CD25 PerCP, anti-ROR*γ*t PE, and Mouse Regulatory T cell Staining Kit, purchased from BD Biosciences (Germany). Enzyme-linked immunosorbent assay (ELISA) kits were purchased from Bioassay Technology Laboratory (China). The DNA extraction kit, viral RNA kit, and RNeasy mini kit were obtained from Qiagen (Germany). cDNA synthesis kit/reagents were purchased from Dena Zist Asia (Iran).

### 2.2. Animals

Forty-eight male Sprague-Dawley rats weighing about 330 g per animal were purchased from Ilam University (Iran). All experimental procedures were conducted in accordance with the International Ethical Standards for the Use and Care of Laboratory Animals. The experimental protocol was approved by the ethical committee at the Ilam University of Medical Sciences, Ilam, Iran (Permit No. IR.MEDILAM.AEC.1401.005).

### 2.3. Bacterial Isolation and Animal Infection

Clinical isolate of *H. pylori* (cytotoxin-associated gene A (*cagA+*), H.12.5) from bacterial bank of isolates, Ilam University of Medical Sciences, Ilam, was obtained from gastric biopsy specimens from patients with gastric ulcers. H.12.5 has AMX and metronidazole sensitivity and clarithromycin resistance phenotype, genotyping exhibit cagA+ and vacA s1m2 polymorphism. The isolate underwent three times in vivo passages in a rat model to facilitate environmental adaptation [17]. Bacteria were suspended in Brucella broth enriched with 10% fetal bovine serum (FBS) at 37°C in a microaerophilic atmosphere (5% O_2_, 10% CO_2_, and 85% N_2_). For culture in rat model, infection took place by gavage administration of 5 × 10^8−10^ colony-forming unit (CFU)/ml (1 ml/rat/day) on three consecutive days following a 12 h fast. After three weeks, gastric biopsies from three rats were examined for indications of infection by hematoxylin and eosin (H&E) staining.

### 2.4. Study Groups

All animals were used with 45 rats inoculated with *H. pylori*, and three rats were not inoculated and designated as the control group. The *H. pylori* infection was confirmed from three rats selected randomly. The remaining 42 rats were also randomly divided into seven groups (*n* = 6) as presented in Table 1.

Metformin concentration in G4 and G5 was selected based on a previous study [9]. In the Courtois et al. study, the metformin dose based on human dosing was 10 mg/mouse/day (the maximum weight for Balb/c is approximately 32 g), which we converted for the in vivo study (in this study, the average weight of the animals was 330 g). In the in vivo pilot study, no antibacterial activity was observed at 100 mg/rat/day, so we tested a double metformin dose of 200 mg/rat/day. The concentration of AMX was determined based on antibiotic therapy in humans. The maximum concentration of AMX in antibiotic therapy against *H. pylori* is about 2 g/day in a human weighing 73 kg (27 mg/kg). We chose a minimum and a maximum AMX concentration for the in vivo study of 10 mg/kg and 20 mg/kg, respectively.

All experimental groups received the drug orally by gavage once daily for two weeks during the experiment. The doses were administered based on previous studies in which metformin has demonstrated anti-inflammatory and *H. pylori*-fighting properties [9, 18, 19]. On day 14 (posttreatment), the rats were euthanized by CO_2_ asphyxiation. An aseptically removed stomach was opened along with its greater curvature, washed in PBS, and divided lengthwise into two parts [20, 21]. One part of the stomach was embedded in paraffin after being fixed in 10% formalin solution according to generally accepted procedures. The second part was used to perform in vivo antibacterial evaluation as described in the following section.

### 2.5. Antibacterial Activity

#### 2.5.1. In Vitro Antibacterial Activity

In vitro antibacterial evaluation was performed using growth curves and colony counts. *H. pylori* was suspended in Brucella broth at 1 × 10^9^ CFU/ml. Optical density (OD) was used to determine the growth curves as an indicator of the antibacterial activity of metformin in the broth at different concentrations (0.1, 0.2, 0.5, and 1.0 M). A 96-well plate containing 150 *μ*l Brucella broth and 50 *μ*l bacterial suspension was incubated at 37°C. As a measure of bacterial growth, turbidity was measured at 600 nm using an ELISA microplate reader at various time intervals (4, 8, 12, 16, 20, and 24 h). Growth curves between the OD value and time were plotted for each test medium. The growth inhibition (GI) was calculated using following formula:

(1) $$\begin{array}{c} \text{GI }\left(\%\right)=\frac{{\text{OD}}_{\text{c}}-\text{O}{\text{D}}_{\text{t}}}{{\text{OD}}_{\text{c}}}\times 100, \end{array}$$

where OD_c_ and OD_t_ represent the OD value of growth curves of the control and the metformin, respectively.

The minimum inhibitory concentrations (MICs) of metformin and AMX were determined by an external test tube method. All experiments were performed in triplicate. MIC was measured for metformin and AMX to assess bacterial susceptibility. After overnight incubation, the MIC of an antibiotic can be defined as the lowest concentration required to inhibit visible bacterial growth. Briefly, H.12.5 was obtained from its frozen stock, grown overnight in the Mueller-Hinton (MH) broth, and then diluted to 10^6^ CFU/ml in the culture medium before use. For determination of MIC, 50 *μ*l of 2-fold diluted metformin (at a concentration of 500 *μ*M) was added to 50 *μ*l of the bacterial suspension in midlog phase on 96-well plates and incubated at 37°C for 18 h to determine MIC. The positive control contained medium and bacterial suspension, while the negative control contained no bacterial suspension. MIC was determined using an ELISA microplate reader at a wavelength of 600 nm.

#### 2.5.2. Synergistic Antibacterial Activity

The synergistic effect of AMX and metformin on *H. pylori* was investigated using a checkerboard assay. We diluted both agents horizontally and vertically in 96-well microtiter plates. Each well was incubated overnight at 37°C with 50 *μ*l bacterial suspension (1.5 × 10^8^ CFU/well), 25 *μ*l metformin (1 M), and 25 *μ*l AMX. After 18 h, MIC was measured using an ELISA microplate reader at 600 nm. All experiments were performed in triplicate. The fractional inhibitory concentration (FIC) of the combination of metformin and AMX was calculated as follows:

(2) $$\begin{array}{c} \text{FIC metformin}=\frac{\text{MIC of metformin in combination}}{\text{MIC of metformin alone}}, \end{array}$$

(3) $$\begin{array}{c} \text{FIC AMX}=\frac{\text{MIC of AMX in combination}}{\text{MIC of AMX alone}}, \end{array}$$

(4) $$\begin{array}{c} \sum \text{F}\text{IC}=\text{FIC metformin}+\text{FIC AMX}. \end{array}$$

The synergism, additive, and antagonistic effects were determined when *Σ*FIC ≤ 0.5, 0.5 < *Σ*FIC ≤ 4, and *Σ*FIC > 4, respectively.

#### 2.5.3. In Vivo Antibacterial Activity

Colony counts were done to evaluate the in vivo antibacterial efficacy. After 24 h incubation, the CFU log of bacterial growth was calculated. Counts were used to calculate the number of surviving bacteria to establish the survival level (SL) and calculated as follows:

(5) $$\begin{array}{c} \text{SL }\left(\%\right)=\frac{C_{\text{c}}-C_{\text{t}}}{C_{\text{c}}}\times 100, \end{array}$$

where *C*_c_ and *C*_t_ represent the count of the control and treatment groups, respectively. After two weeks of treatment, gastric biopsies were carried out. One gram of the stomach was weighed, carefully homogenized, and soaked in 2 ml of PBS. Later, 0.5 ml of the suspension was dispensed onto selective blood agar medium and supplemented with human blood (10% *v*/*v*) and antibiotics (10 *μ*l/ml vancomycin, 5 *μ*l/ml trimethoprim, and 1 *μ*l/ml amphotericin B) at 37°C for 72 h under microaerophilic conditions. Bacteria based on biopsies from each group were stained with the Giemsa and immunohistochemistry (IHC) and counted microscopically.

#### 2.5.4. Weight Change Observation

The animal weights in the three groups, NS, Met-1, and Met-2, were regularly determined and compared until the completion of the experiments.

### 2.6. RT-PCR

Based on the pathological effect of the *cagA* gene of *H. pylori* and the secretion of the cytokine IL-8, we investigated the effect of metformin on the gene expression of *cagA* and IL-8 in vivo by RT-PCR. Total RNA was extracted with RNeasy mini kits and reverse transcribed into cDNA. The *cagA*+ *H. pylori* was used as a positive control and the HEK-293 cell line as a negative control. Glyceraldehyde-3-phosphate dehydrogenase (GAPDH) and *ureC* were used as internal controls. Each reaction consisted of a total volume of 25 *μ*l when combined with reagents from the TaKaRa Ex Taq® Kit (Takara) and stained with SYBR green. The primers are listed in Table 2. GAPDH and *ureC* were used to normalization of IL-8 and cagA, respectively. Each reaction consisted of a total volume of 25 *μ*l when combined with reagents from the TaKaRa Ex Taq® Kit (Takara) and stained with SYBR green. Thermocycling conditions were 35 cycles of denaturation at 95°C for 1 min, annealing at 60°C (for IL-8 and GAPDH) and 50°C (for *cagA* and *ureC*) for 30 s, and extension at 72°C for 1 min. All samples were assessed in triplicate.

### 2.7. Lymphocyte Isolation and Flow Cytometry

Lymphocytes were isolated from the spleens of experimental rats. To examine Treg cells, 5 × 10^5^ cells were stained with anti-CD4 and anti-CD25, followed by intracellular anti-FOXP3, according to the manufacturer's protocol, at 2-8°C for 40-50 min in the dark. For the detection of Th17 cells, cells were stained with anti-CD4 and intracellular anti-ROR*γ*t.

After the rats were sacrificed on day 14, their spleens were removed from the abdominal cavity and sufficiently minced. They were then filtered through a 40 *μ*m filter to prepare for the suspensions of splenocytes. The erythrocytes in the spleen suspension were lysed by lysis buffer. Splenocytes were then stained with mAbs. For intracellular staining, cells were incubated with mAbs for 45 min at 4°C in RPMI 1640 medium containing 5% FBS. Flow cytometry was performed using a FACSCalibur flow cytometer (BD Biosciences, USA), and the corresponding data were analyzed using FlowJo software (Tree Star, Inc., Ashland, OR, USA).

### 2.8. Enzyme-Linked Immunosorbent Assay

A whole blood sample was taken from the rats. The presence of IL-1*β*, TGF-*β*, IL-10, IL-6, and IL-17A in serum was measured using ELISA kits according to the manufacturer's instructions. The intensity was determined at 450 nm using a microplate reader. To ensure consistency of the assay, all plates contained positive control (FBS) and negative control (PBS) samples.

### 2.9. Histologic Examination

After treatment and dehydration of the tissues, paraffin embedding was performed. Samples of 4 *μ*m thick were excised from the blocks perpendicular to the mucosal surface. Sections for histological analysis were taken on glass slides. The Giemsa staining was used to detect the presence of *H. pylori* in the paraffin sections of preserved gastric tissue. A classification system was developed to determine the degree of bacterial colonization-values of eradication: no bacterial detection (-); mild, colonization in some gastric crypts (+); moderate, colonization in all gastric crypts (++); and severe, colonization in all gastric crypts (+++).

Infiltration of polymorphonuclear neutrophils (PMNs) and mononuclear inflammatory cells (MNCs) in the gastric mucosa and atrophy was scored from 0 to 3 plus, (0, 1, 2, and 3, corresponding to none, mild, moderate, and severe, respectively) [22]. An independent pathologist evaluated the histology of the stomach, and the results were expressed as the mean of the scores for each group [23].

### 2.10. Statistical Analysis

The results were statistically analyzed using *t*-test with a confidence level of 95% (*P* < 0.05). Differences between means were tested for more than two groups using one-way analysis of variance (ANOVA) followed by multiple comparisons with the Bonferroni correction. Differences between groups and time points were analyzed with a two-way ANOVA and subsequent multiple comparisons using the Bonferroni correction. ^∗^*P* < 0.050, ^∗∗^*P* < 0.001, ^∗∗∗^*P* = 0.0002, and ^∗∗∗∗^*P* < 0.0001 indicated statistically significant results. All statistical analyses were performed in GraphPad Prism eight software. The *P* value of less than 0.05 was considered as significant.

## 3. Results

### 3.1. Antibacterial Activity

**Figure 1:**
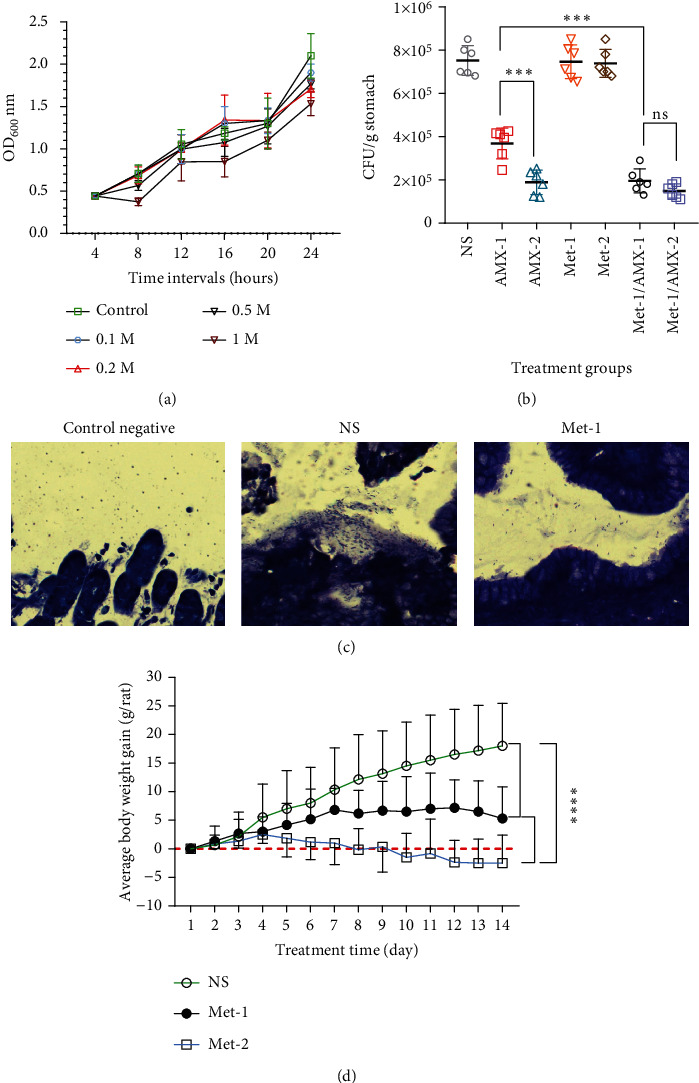
Metformin reduces the effective dose of AMX against *H. pylori*. (a) Growth curve of metformin at different concentrations (0.1, 0.2, 0.5, and 1 M) at regular intervals (4, 8, 12, 16, 20, and 24 h). (b) In vivo antibacterial activity of metformin alone and in combination with AMX. (c) The distribution of *H. pylori* on the biopsy section, Giemsa's staining. (d) Metformin significantly reduced body weight at two concentrations (100 and 200 mg/kg) for two weeks compared with the control group (which received normal saline). The values are presented as the mean and the standard error of the mean (SEM). ^∗^*P* < 0.05, ^∗∗^*P* ≤ 0.01, ^∗∗∗^*P* ≤ 0.001, and ^∗∗∗∗^*P* < 0.0001.

Metformin showed anti-*H pylori* activity at a concentration of 1.0 M. The difference between 16 h and 24 h time intervals was statistically significant (*P* = 0.01 and *P* = 0.04, respectively) (Figure 1(a)). Statistical analysis revealed a significant difference between metformin's antibacterial activity at examined concentrations at the following time intervals: 1.0 M vs. 0.1 M and 0.2 M at the 16 h interval (*P* = 0.009 and *P* = 0.003, respectively) and 1.0 M vs. control and 0.2 vs. control at the 24 h interval (*P* = 0.0006 and *P* = 0.03, respectively).

The MIC of metformin was >500 *μ*M, whereas the MIC of AMX was 10.68 *μ*M. A moderate decrease in the MIC of the antibiotic was observed when metformin was taken together with AMX. The combination of metformin and AMX resulted in a decrease in AMX MIC of approximately 24% to 2.61 *μ*M. In addition, a strong reduction in the MIC of metformin was observed, and the MIC in combination was reduced by 50 *μ*M. FIC indices were calculated, and a synergistic effect was observed (*Σ*FIC = 0.34) (Table 3).

An evaluation of the in vivo anti-*H. pylori* potential of each group was performed using biopsies. In all control rats treated with NS, the *H. pylori* concentration in the stomach remained at a level of approximately 7 × 10^5^ CFU/g stomach. In all groups receiving AMX, there was a significant reduction in CFU compared to those receiving metformin and control. As a single agent, metformin showed no anti-*H. pylori* activity in vivo, whereas in combination with AMX, it reduced CFU counts. We selected 100 mg/kg metformin for further in vivo studies in combination with antibiotics. It was found that rats receiving metformin in combination with AMX (Met-1/AMX-1) had significantly lower CFU counts than the AMX-1 group (*P* = 0.001) (Figure 1(b)). It was observed that the AMX-treated groups exhibited significantly different levels of antibacterial activity (*P* = 0.003), while AMX at a dose of 10 mg/kg showed antibacterial activity close to that of 20 mg/kg in the presence of metformin. These results were confirmed by histologic evaluation of the biopsies (Figure 1(c)). A comparison of weight loss between the metformin-treated group and the control group is shown in Figure 1(d).

### 3.2. Comparison of IL-8 mRNA Expression with the Level of cagA Expression of Colonized H. pylori

The pathogenicity of *H. pylori* is mediated by a number of virulence factors. The oncoprotein encoded by *cagA*, an eminent virulence factor, is involved in the elongation of epithelial cells and the production of inflammatory cytokines such as IL-8 [24].

**Figure 2:**
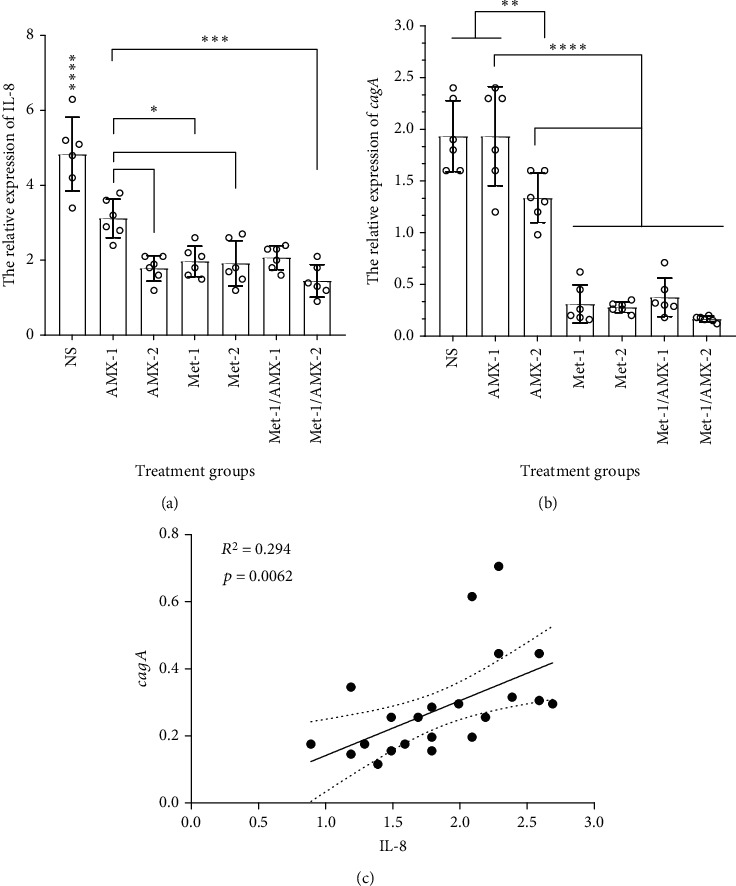
The relative expression of (a) IL-8 and (b) cagA. (c) Regression analysis of the relationship between the expression of IL-8 and cagA. Values are shown as mean and SD. *N* = 6.

The relative expression of IL-8 was compared with the expression of *cagA* to investigate the effects of metformin on inflammation-mediated cagA and the associated cytokine IL-8. As compared to the control group, metformin significantly reduces the expression of IL-8 (*P* < 0.0001) (Figure 2(a)). A significant increase in the expression of IL-8 was observed in AMX-1 compared to the other treated groups. A significant difference was also observed between AMX-1 and AMX-2 (*P* = 0.04). Levels of relative expression of IL-8 were found to be lower in the metformin-treated groups than in the AMX-1 group. Metformin significantly reduces the expression of *cagA* compared to the control groups (Figure 2(b)). Statistical analysis showed that increased cagA expression in the same sample was associated with a high level of IL-8 (Figure 2(c)). Accordingly, the decrease of cagA expression results in reduction of IL-8 expression in tissue.

### 3.3. Metformin Mediates a Treg-Related Response

**Figure 3:**
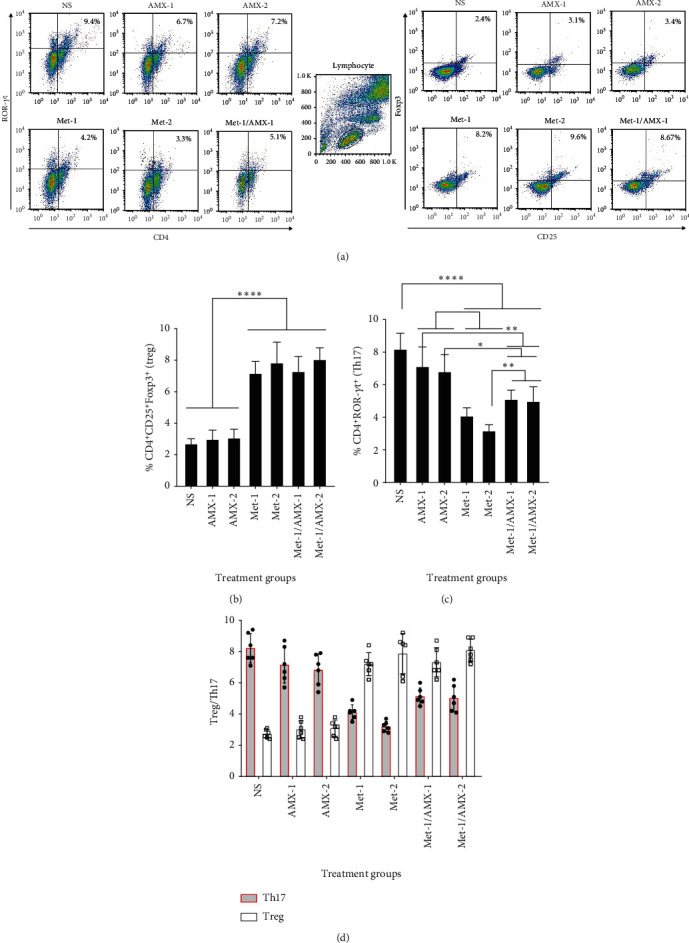
Compared with AMX administration, metformin administration shifts the Treg/Th17 ratio toward a population with a higher number of Treg cells. (a) Flow cytometry characterization shows positive expression of ROR*γ*t^+^ (left panel) and coexpression of CD25^+^ and Foxp3^+^ (right panel) among CD4^+^ T cells for Th17 and Treg cells, respectively. (b) Comparison of the percentage of Th17 cells in the different treatment groups. (c) Comparison of the percentage of Treg cells in the different treatment groups. (d) Treg/Th17 ratio in the different treatment groups. Values are shown as mean and SD. *N* = 6.

To assess Treg-mediated immunological response to *H. pylori* colonization, we determined the percentage of CD4+ ROR*γ*t+ Th17 cells and CD4+CD25+FOXP3+ Treg cells in the spleens of model rats (Figure 3(a)). In contrast to metformin, which significantly decreased the proportion of Th17 cells, there was no discernible difference in the amount of these cells between the AMX and control groups two weeks after treatment (Figures 3(a) and 3(b)). The results showed that the presence of metformin was responsible for the reduction of TH17 cells in the combination groups. The prevalence of Th17 cells decreased significantly when AMX and metformin were administered in combination compared with AMX alone (Figure 3(b)). On the other hand, rats receiving metformin showed a significant increase in the proportion of Treg cells (Figures 3(a) and 3(c)). In the groups receiving AMX, the balance between Treg and Th17 cells did not differ from the control groups, whereas metformin administration resulted in a change in the Treg/Th17 ratio (Figure 3(d)).

### 3.4. The Profile of Treg/Th17-Related Cytokines

**Figure 4:**
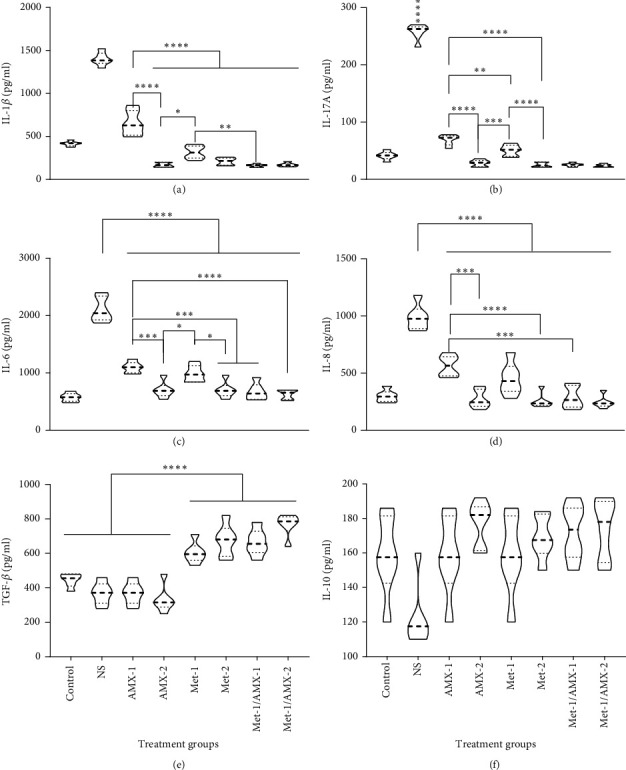
The concentrations of Th17- and Treg-related cytokines in serum samples at the end of the experiments in different groups including control, NS, AMX-1, AMX-2, Met-1, Met-2, Met-1/AMX-1, and Met-1/AMX-2. Serum levels of cytokines including (a) IL-1*β*, (b) IL-17A, (c) IL-6, (d) IL-8, (e) TGF-*β*, and (f) IL-10 were determined by ELISA. The above data represent the mean ± SD of triplicate measurements. *N* = 6.

**Figure 5:**
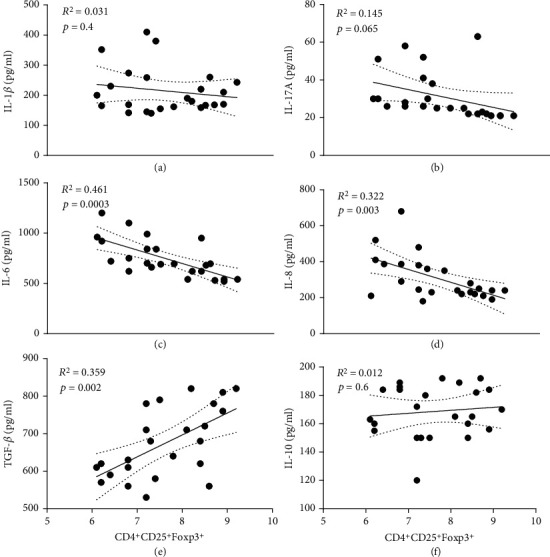
The correlation between the number of Treg cells and the cytokine levels assessed. Linear regression analysis of Treg cells with the level of cytokines including (a) IL-1*β*, (b) IL-17A, (c) IL-6, (d) IL-8, (e) TGF-*β*, and (f) IL-10 was performed. *N* = 6.

By analyzing serum levels of cytokines associated with Tregs and Th17 cells, we evaluated the effect of metformin on cytokine profiles after alteration of Treg/Th17 balance and compared the results with those of the control groups. As shown in Figure 4, the levels of IL-1*β*, IL-17, IL-8, and IL-6 in the serum of rats at the end of the experiment showed a significant decrease in all treatment groups. A significant change in cytokine profile occurred during AMX administration in a dose-dependent manner. The concentration of IL-1*β* was significantly lower in the AMX-2 group than in the AMX-1 and Met-1 groups (Figure 4(a)). The combination of AMX with metformin significantly reduces IL-1*β*, IL-6, and IL-8 (Figures 4(a), 4(c), and 4(d)). The results indicate that metformin seems to be responsible for the reduction of these cytokines. The reduction of Th17-related cytokines such as IL-6 and IL-17 was associated with the concentration of metformin (Figure 4(b)). AMX had a stronger effect on the concentration of all Th17-related cytokines when administered at a higher dose than when administered at a lower dose. The elimination of *H. pylori* may be responsible for the reduction in Th17-related cytokines. The results suggest that metformin administration significantly increases the concentration of TGF-*β*, an important Treg-related cytokine (Figure 4(e)). IL-10 was slightly higher in all treatment groups than in the control group (Figure 4(f)). Statistical analysis of the metformin-containing groups, including the alone and combined groups, showed that a negative correlation was found between the inflammatory cytokines IL-6 and IL-8 and the percentage of Treg cells, whereas a positive correlation was found between the increase in Treg cells and TGF-*β* levels (Figures 5(a)–5(f)).

### 3.5. Histological Evaluation

**Figure 6:**
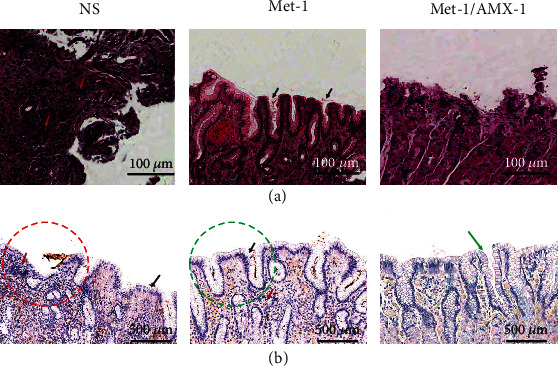
Histological evaluation, (a) H&E and (b) IHC staining of biopsies, the NS groups showed severe chronic superficial gastritis with chronic inflammatory cells including polymorphonuclear and mononuclear cells (red circle and red arrow), the Met-1 group showed typical mild colonization of *H. pylori* in the antrum pits with reduced infiltration of immune cells (green circle and black arrow), and the Met-1/AMX-1 group showed bacterial clearance (green arrow) and very low infiltration of immune cells.

Bacterial colonization results and histopathological scores are presented in Table 4. During combination therapy, the bacterial colonization was significantly lower in the mucosa of the Met-1/AMX-1 groups, whereas was mild in the AMX-1 groups. Pathological evaluations showed that the presence of metformin reduced tissue damage and inflammation levels compared with the control group, whereas lymphocyte infiltration was significantly reduced and bacterial colonization was low in the combined group (Met-1/AMX-1) (Figure 6(a)).

Inflammation was reduced in response to metformin alone and plus AMX, whereas AMX powder had no anti-inflammatory effect. Bacterial colonization was significantly reduced during AMX administration, whereas it was high in the groups receiving metformin. Inflammation scores and infection density were lower in the AMX-2 group than in the other AMX groups. Immune cell infiltration was greater in the groups receiving metformin than in the groups receiving AMX (Figure 6(b)).

## 4. Discussion

The development of *H. pylori* infection and its side effects, e.g., gastritis, are closely related to changes in the balance of Treg/Th17 cells. This helps to better understand the pathophysiology of the disease and to find new treatment targets [25]. In the present study, the effects of metformin on immune regulation and antibacterial activity were investigated in detail in a rat model of *H. pylori* infection. Metformin has potential activity against *H. pylori*, but there is ample evidence of its synergistic effect with conventional antibiotics against Gram-positive and Gram-negative bacteria [5, 9, 26–28]. Previously, Courtois et al. reported that 10 mg/mouse/day of metformin had antibacterial activity against *H. pylori* [9]. In this study, we investigated the antibacterial activity of metformin at the same concentration but found no antibacterial activity. Antibacterial activity can be controversial due to the differences in strains and animal models.

Our study shows that the combination of metformin with AMX has a strong synergistic effect against *H. pylori* infection. This effect was also confirmed in vivo. The antibacterial results in vivo showed that the combination of the two aforementioned agents can reduce the effective dose of the antibiotic (Figure 1(b)). Some studies have been performed to reduce the MIC of AMX to avoid antibiotic pressure. For example, Attia et al. found that administration of zinc oxide nanoparticles together with AMX lowered the MIC90 of the antibiotic. The synergistic effect of zinc oxide was found when gall extract was added [29]. In a study by Masadeh et al., metformin was presented as a potential adjuvant in antibiotic therapy against Gram-negative and Gram-positive bacteria. Metformin in combination with various antibiotics, including levofloxacin, chloramphenicol, rifampicin, ampicillin, and doxycycline, was tested against methicillin-resistant *Staphylococcus aureus* and *Pseudomonas aeruginosa* (*P. aeruginosa*, ATCC BAA-2114) as multidrug-resistant strains to determine their synergistic effect. Masadeh et al. found that metformin in combination with all antibiotics tested showed a synergistic effect against *P. aeruginosa*. The results were the same for MRSA, except that the combination of metformin and rifampicin showed an additive effect [6]. Recently, Khayat et al. found that the combination of metformin and vildagliptin had an anti-quorum-sensing effect on *P. aeruginosa* infections. The quorum-sensing-blocking concentration of the components was much lower than clinical use, 10 and 1.25 mg/ml versus 850 and 50 mg/ml, respectively [30]. According to microbiological studies, metformin is a reliable adjuvant to antibiotic agents against Gram-negative and Gram-positive bacteria in infections and biofilm formation. In this study, we show that metformin reduces the pathogenicity of *cagA*+ *H. pylori* to cause gastric ulcers. However, metformin can be used as an adjuvant to reduce the effective dose of AMX. We have previously reduced the effective dose of this antibiotic by encapsulation with chitosan and docosahexaenoic acid [31].

A previous study showed that *H. pylori* shifts the balance between Th17 and Treg cells in favor of Treg cells and inhibits responses associated with Th17 cells [25]. Another study suggests that *H. pylori* inflammation and colonization in the stomach may be regulated by CD4+FOXP3+ Treg cells [32]. There is also evidence that *H. pylori* expresses virulence factors (e.g., *cagA*) after colonization of the gastric glands and elicits immune responses mediated by T cells that lead to destruction of the epithelium and cause a strong inflammatory response, namely, gastritis [16]. In the study by Raghavan et al., *H. pylori* was able to colonize the stomach of nude mice without causing tissue damage or inflammation [13]. Treg cells are thought to limit inflammatory responses to *H. pylori* by producing anti-inflammatory cytokines such as IL-10 and TGF-*β*. This process is positively correlated with chronic gastritis and adenocarcinoma of the stomach, which may lead to persistence of the pathogen [16].

Metformin is used for its safety and curative properties in various diseases, including diabetes and asthma [33]. A previous study showed that in nonobese diabetic mice treated with metformin, the development of autoimmune insulitis significantly decreased and the number of proinflammatory IFN-*γ*+ and IL-17+ CD4 T cells in the spleen was significantly reduced [3]. In our study, metformin administration shifted the Treg/Th17 balance in favor of Treg cells, and proinflammatory cytokines (IL-17A, IL-6, and IL-8) and Treg-mediated cytokines (TGF-*β*) also significantly decreased and increased, respectively. In addition, AMX administration showed no immunoregulatory effect when the Treg/Th17 ratio was considered. In the study by Locke et al., no association was found between AMX administration and increased Treg frequency [34].

Patients with *cagA*-positive *H. pylori* have been shown to have severe gastritis in contrast to those with *cagA*-negative *H. pylori* [35]. CagA expression can be both beneficial and detrimental to *H. pylori* survival in the stomach. CagA can cause loss of tight junctions and polarity of epithelial cells by disrupting zona occludens-1 and adhesion molecules. It can also induce inflammation in gastric epithelial cells by expressing proinflammatory cytokines [36]. In vitro, *H. pylori* strains expressing CagA induce high IL-8 secretion in gastric epithelial cells [37].

The development of muscle atrophy, intestinal metaplasia, and high-grade inflammation is considered to be different effects of infection with *cagA*-positive strains. In addition, this infection is associated with overexpression of proapoptotic proteins in the gastric mucosa. CagA is an important pathogenic factor that mainly causes tissue damage and severe gastritis. Therefore, it is likely that metformin could affect the expression of virulence factors, especially CagA and its associated cytokine IL-8 [38]. A previous study reported that the levels of cagA and IL-8 were lower in the group receiving metformin than in the control group and in the groups receiving AMX. Although the mechanism of action of metformin is unknown, it appears that this decrease occurs during the induction of immune tolerance to bacteria by metformin [13]. However, one study has shown that the expression of *cagA* is higher in the gastric mucosa of mice infected with *H. pylori* with a less severe immune response than in mice with a more severe immune response [36]. In mice lacking Treg cells, *H. pylori* burden is lower and gastritis is more severe [13]. The results show that metformin increases the number of Tregs, suggesting that it effectively reduces gastritis but increases *H. pylori* survival. On the other hand, the combination of AMX with metformin may lead to a decrease in *H. pylori*. It has been shown that taking metformin against *H. pylori* is harmful to the patient and may be responsible for the development of gastric cancer [1].

In the groups of mice receiving metformin, tissue damage was associated with a decrease in *cagA* and proinflammatory cytokines. The results also showed that metformin treatment resulted in immune tolerance to *H. pylori* infection. In addition, clearance of *H. pylori* was significantly higher in groups treated with a combination of AMX and metformin than in groups treated with these agents alone. Raghavan et al. recently pointed out that Treg cells reduce immunopathology in *H. pylori* infection despite a higher *H. pylori* burden in the gastric mucosa [13]. Recently, however, metformin was shown to be a potential drug against gastric cancer [1]. Shuai et al. reported that the use of metformin reduced the incidence of gastric cancer. In Asian patients with type 2 diabetes, the use of metformin reduces the risk of gastric cancer compared with Western patients. Meta-analysis showed that metformin use increased overall survival and cancer-specific survival [39]. The role of metformin in GC prevention may be undermined by the fact that previous studies did not adequately consider other important risk factors, such as *H. pylori* infection and severity of diabetes mellitus [1, 39, 40]. Because GC is primarily caused by *H. pylori*, it will be difficult to accurately assess the effects of metformin on the development of GC unless patients are stratified by *H. pylori* status. In addition, it is important to properly diagnose *H. pylori* infection before starting metformin treatment, as this may increase the risk for certain adverse effects. However, metformin therapy in GC patients is significantly affected by the status of *H. pylori* infection. According to a study by Cheung et al., metformin use in diabetic patients with *H. pylori* eradication was associated with a lower risk of GC [41]. Thus, *H. pylori* eradication suggests a critical role of *H. pylori* infection in the outcomes of metformin therapy for gastric cancer. Future studies should investigate the role of metformin in reducing the risk of GC in patients with *H. pylori* infection. In addition, further research should be conducted to determine the optimal dosage and frequency of metformin administration for GC prevention. Several studies have shown that metformin has various biological effects against cancer, with cell cycle inhibition being one of the most important. Kato et al. found that metformin inhibited the proliferation of human gastric cancer cell lines such as MKN1, MKN45, and MKN74. Alteration of microRNA expression, decrease of C1 cyclins, and phosphorylation of epidermal growth factor receptor and insulin-like growth factor-1 receptor were responsible for cell cycle inhibition of all cell lines [42]. Gastric cancer stem cells (CSCs) are characterized by self-renewal and asymmetric division properties and by their ability to generate tumor cells with different phenotypes. One study showed that metformin induced cell cycle arrest that reduced cell proliferation and decreased the number of tumor spheres, illustrating its ability to target CSCs [43]. In a recent study by Kao et al., metformin inhibited gastric cancer cell growth and induced cell apoptosis. This effect was evident when metformin was used in combination with lansoprazole, a proton pump inhibitor [44]. Metformin induced apoptosis and cell cycle arrest in part by inhibiting PARP expression. Metformin downregulated the expression of PI3K, Akt, HIF1*α*, PARP, PKM2, and COX. In addition, overexpression of HIF1*α* increased viability, invasion, and migration of gastric cancer cells [45]. Notwithstanding the controversy, metformin appears to be an effective drug in the treatment of patients with gastric cancer, although there is probably a difference between growth inhibition and apoptosis and immune regulation in the anticancer effects of metformin.

## 5. Conclusions

Metformin is an inexpensive and well-tolerated oral agent commonly used as first-line therapy for type 2 diabetes. It has been extensively studied as an antibacterial and anti-inflammatory agent for potential treatment. In this study, we found that the combination of metformin and AMX had a strong synergistic effect. *H. pylori* pathogenicity decreased when the balance between Treg and Th17 cells was shifted toward Treg dominance. While metformin decreased early *H. pylori* complications, particularly gastritis, bacterial colonization persisted. Metformin administration reduced *H. pylori*-related inflammation by inducing Tregs, and after using metformin in combination with an antibiotic, i.e., AMX, the effective dose of antibiotics for complete eradication of *H. pylori* decreased.

## Figures and Tables

**Table 1:** The design of experimental groups and treatment options.

Groups	Name	Treatment drugs
G1	Normal saline (NS)	Normal saline (1 ml)
G2	AMX-1	10 (mg/kg)
G3	AMX-2	20 (mg/kg)
G4	Met-1	100 (mg/rat/day)
G5	Met-2	200 (mg/rat/day)
G6	Met-1/AMX-1	Met (100)+AMX (10)
G7	Met-1/AMX-2	Met (100)+AMX (20)

**Table 2:** Primer sequences for qRT-PCR.

Primer name	Sequences
cagA	F-5′GATAACAGGCAAGCTTTTGAGG3 ′ R-5′CTGCAAAAGATTGTTTGGCAGA3 ′
ureC	F-5′GGATAAGCTTTTAGGGGTGTTAGGGG3 ′ R-5′GCTTGCTTTCTAACACTAACGCGC3 ′
IL-8	F-5′TCAGAGACAGCAGAGCACAC3 ′ R-5′GGCAAAACTGCACCTTCACA3 ′
GAPDH	F-5′ATTCCACCCATGGCAAATTC3 ′ R-5′GCATCGCCCCACTTGATT3 ′

**Table 3:** Antibacterial activity of metformin and AMX against *H. pylori*.

	Metformin	AMX
MIC alone	>500 *μ*M	10.68 *μ*M
MIC in combination	50 *μ*M	2.61 *μ*M
FIC	0.1	0.24
*Σ*FIC	0.34	

**Table 4:** Bacterial colonization and histological findings.

	Bacterial colonization^∗^	Ulceration/necrotic tissue	Inflammatory cell infiltrates	Congested blood vessels	Edema
NS	+++	+++	+++	++	++
AMX-1	+	++	++	+	++
AMX-2	-	++	+	+	+
Met-1	+++	+	+	-	+
Met-2	+++	-	+	-	+
Met-1/AMX-1	-	-	+	-	-
Met-1/AMX-2	-	-	+	-	-

^∗^To determine bacterial colonization, one biopsy was obtained from each sample.

## Data Availability

The data that support the findings of this study are available from the corresponding author upon reasonable request.
